# Numerical Study of Turbulent Pulsatile Blood Flow through Stenosed Artery Using Fluid-Solid Interaction

**DOI:** 10.1155/2015/515613

**Published:** 2015-08-31

**Authors:** Mehdi Jahangiri, Mohsen Saghafian, Mahmood Reza Sadeghi

**Affiliations:** ^1^Department of Mechanical Engineering, Isfahan University of Technology, Isfahan 8415683111, Iran; ^2^Department of Biomedical Engineering, University of Isfahan, Isfahan 8174673441, Iran

## Abstract

The turbulent pulsatile blood flow through stenosed arteries considering the elastic property of the wall is investigated numerically. During the numerical model validation both standard *k*-*ε* model and RNG *K*-*ε* model are used. Compared with the RNG *K*-*ε* model, the standard *K*-*ε* model shows better agreement with previous experimental results and is better able to show the reverse flow region. Also, compared with experimental data, the results show that, up to 70% stenosis, the flow is laminar and for 80% stenosis the flow becomes turbulent. Assuming laminar or turbulent flow and also rigid or elastic walls, the results are compared with each other. The investigation of time-averaged shear stress and the oscillatory shear index for 80% stenosis show that assuming laminar flow will cause more error than assuming a rigid wall. The results also show that, in turbulent flow compared with laminar flow, the importance of assuming a flexible artery wall is more than assuming a rigid artery wall.

## 1. Introduction

Atherosclerosis is a disease that is characterized by the formation of plaques that narrow the arterial lumen. The narrowing of the coronary arteries can stop the perfusion of blood to the lower parts of the myocardium and possibly lead to myocardial ischemia, myocardial infarction, and sudden cardiac death [1].

In the study of the causes and progression of this disease, in addition to conventional methods in the medicine for forecasting and evaluating the disease progress, computational fluid dynamics are used to examine the role of hemodynamics on the localization, development, and progression of atherosclerosis disease. Simultaneous with hemodynamic studies, some researchers have focused on modeling the arterial wall and have examined the relationship between arterial wall stress and vessel wall diseases [2–4].

Recently, researchers have paid great attention to the effect of fluid-solid interaction in biological systems, especially cardiovascular ones. They believe that the simultaneous solution of fluid-solid will greatly help in better understanding the pattern of arterial disease [5, 6].

For example, Bathe and Kamm [7] simulated the laminar pulsatile flow passing through a flexible artery with stenosis using ADINA Software. They considered stenoses of 51% and 96% area reduction and evaluated and compared the pressure drop and circumferential stress across the artery at different times. They also studied the effect of Reynolds number on the pressure drop.

Tang et al. [8] numerically examined laminar flow in flexible carotid artery with symmetric stenosis using ADINA software. Their results showed that severe stenosis causes critical flow conditions such as negative pressure and high and low shear stress which may lead to artery compression, plaque rupture, platelet activation, and arterial thrombosis.

Although in such studies the flexibility of the artery wall has been considered, they have ignored the turbulence caused by stenosis. In fact, blood flow in arteries is usually laminar. However, a moderate or severe stenosis can cause turbulent flow in the vasculature [9]. A better understanding of the flow and flow turbulence in the poststenotic region can lead to more accurate diagnostic methods [10]. Turbulent blood flow due to arterial stenosis has long been investigated [11].

Many experimental studies have been conducted for studying a steady turbulent flow [12–14]. Deshpande and Giddens [13] studied the steady turbulent flow through a 75% stenosed tube at Reynolds numbers ranging from 5000 to 15000 by laser Doppler anemometer (LDA). Ahmed and Giddens [14] measured the steady velocity field in the presence of a symmetric stenosis with a rigid wall by the LDA. The range of the Reynolds number was 500–2000 in the upstream of the stenosis and stenoses of 25, 50, and 75% area reduction were studied.

Due to difficulties in performing experimental tests, there are only a few experimental studies for unsteady turbulent flow in the presence of stenosis.

Ahmed and Giddens [15] measured pulsating flow field in the presence of a symmetric stenosis by the LDA. They considered sinusoidal velocity profile, a Womersley number of 7.5, stenoses of 25, 50, and 75% area reduction, and the average Reynolds number of 600 for testing.

These experimental studies showed that, even with a low percentage of stenosis, transient or turbulent flow may occur. The above experimental data were used for assessment of numerical methods for modeling of turbulent flow in internal flows. On the other hand since the turbulent flow calculations are difficult and time-consuming, there are very few computational studies on the turbulent pulsatile flow in the artery with a stenosis. For example, using the finite element software FIDAP, Ghalichi et al. [16] investigated transient and turbulent flow through 50%, 75%, and 85% stenosed models over a Reynolds number range of 500 to 2000. Their results showed that the laminar flow model overestimates the vortex length when the flow becomes transitional or turbulent.

Banks and Bressloff [17] modeled pulsatile turbulent flow in the carotid bifurcation with a stenosis by a three-dimensional model. FLUENT software was used for solving the set of governing equations. Three types of stenosis (mild, moderate, and severe) were considered, and the effect of turbulence intensity and turbulent viscosity on velocity profiles was studied.

Since wall elastic property and physiological pulses are not considered as boundary conditions in these studies, in the present study the turbulent blood flow through a stenotic artery model is numerically simulated considering fluid-structure interaction (FSI) using ADINA 8.8. At first the effect of turbulent blood flow on the variations of time-averaged shear stress and the oscillatory shear index for 80% stenosis is investigated. Then the obtained results are compared with the results of assuming laminar flow and rigid wall of coronary artery.

## 2. Governing Equations

### 2.1. Reynolds-Averaged Navier–Stokes Equations (RANS) [18]

In unsteady turbulent flows, if we consider each parameter as the sum of an average component and an oscillating component in the Navier–Stokes equation, then the RANS equations are obtained as follows:

(1) ∂u−i∂xi0,Du−iDt=−1ρ∂P−∂xi+∂∂xjμ+μTρ∂u−i∂xj+∂u−j∂xi.

### 2.2. Turbulence Models [18]

To calculate *μ*_*T*_, this paper uses the two-equation turbulence *K*-*ε* standard and *K*-*ε* RNG models. In the turbulence flow, viscosity is defined as follows, where *μ*_0_ is laminar viscosity and *μ*_*T*_ is turbulence viscosity:

(2) $$\begin{array}{c} \mu =\mu_{T}+\mu_{0}. \end{array}$$

In *K*-*ε* standard turbulence model, *μ*_*T*_ is calculated as follows:

(3) $$\begin{array}{c} \mu_{T}=\rho c_{\mu }\frac{k^{2}}{\varepsilon }, \end{array}$$

where *k* is turbulence kinetic energy and *ε* is turbulence dissipation rate.

## 3. Numerical Validation

To check the accuracy of our numerical solution, the numerical results of the present work are compared with the experimental results presented by Ahmed and Giddens [15] and the numerical results provided by the Banks and Bressloff [17] and Varghese and Frankel [19].

**Figure 1:**
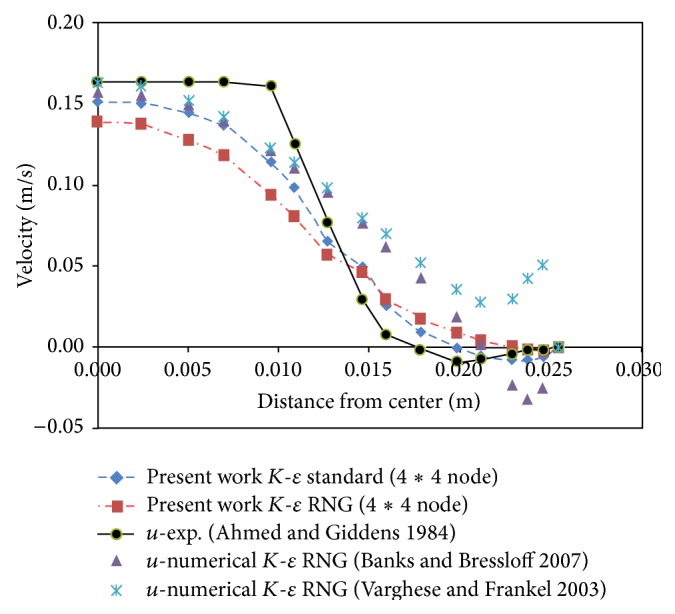
Velocity profile at distance *z* = *D* from throat of stenosis.

**Figure 2:**
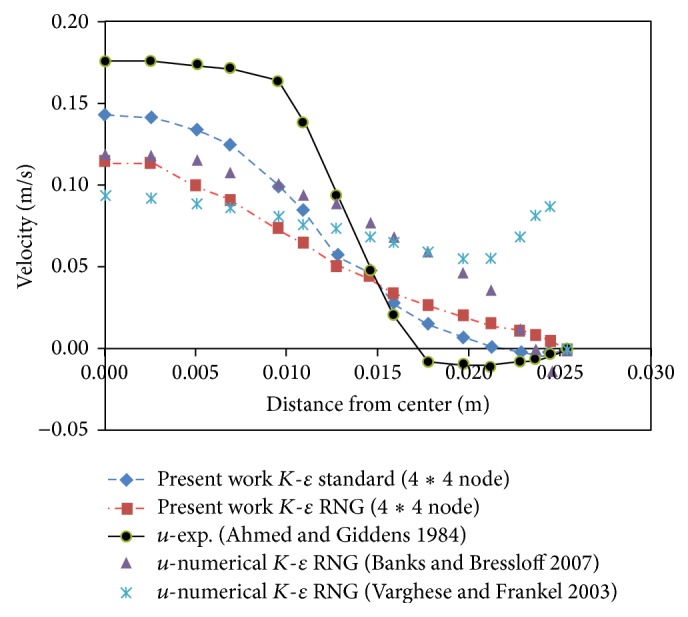
Velocity profile at distance *z* = 1.5*D* from throat of stenosis.

If we consider the origin of coordinates at the center of stenosis, the numerical results of the present work were compared with the experimental results presented by Ahmed and Giddens [15] and the numerical results provided by Banks and Bressloff [17] and Varghese and Frankel [19] at two different distances of stenosis downstream and in the time of maximum speed. The results of Figures 1 and 2 indicate a better agreement of numerical data of the present work with the results of Ahmed and Giddens [15] than the numerical results of Banks and Bressloff [17] and the numerical work of Varghese and Frankel [19].

The results indicate a higher consistency between the *K*-*ε* standard model and experimental results. As a result, the *K*-*ε* standard model was used in this study.

## 4. Present Work and Numerical Methods Used

**Figure 3:**
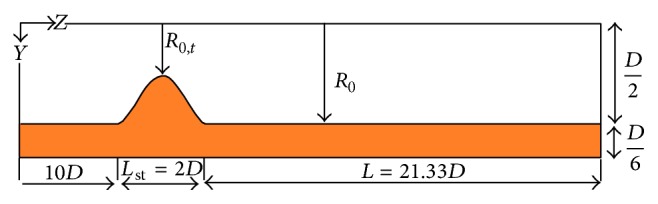
Computational domain.

In this study a model of coronary artery with a simple, symmetrical stenosis with flexible wall is considered. The computational domain and its dimensions are shown in Figure 3.

The geometry of stenosis is defined as follows [21]:

(4) $$\begin{array}{c} \frac{R\left(z\right)}{R_{0}}=1-\left(\;\frac{R_{0}-R_{0,t}}{2R_{0}}\;\right)\left(\;1+\cos\frac{2\pi \left(z-z_{m}\right)}{L_{\text{st}}}\;\right), \end{array}$$

where *R*_0_ is the radius of the healthy artery, *R*(*z*) is the artery radius in the stenosis region, *R*_0,*t*_ is artery radius at the stenosis throat, *z*_*m*_ is the location of the center of the stenosis, and *L*_st_ is the length of stenosis. The characteristics of blood as a Newtonian, incompressible fluid and the characteristics of artery wall are given in Table 1 [21].

**Figure 4:**
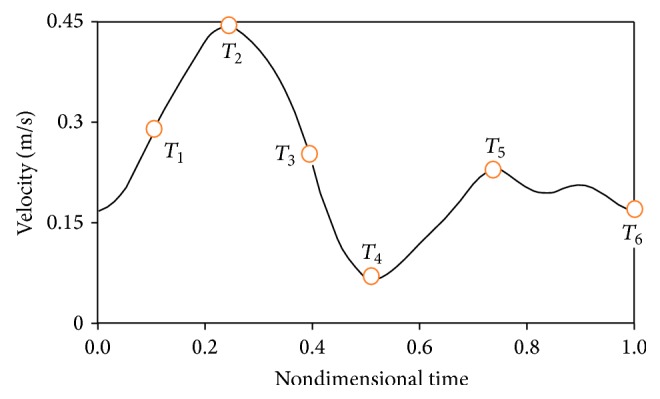
Inlet pulsatile velocity profile [20].

The pulsatile velocity profile of the right coronary artery was used as the inlet boundary condition [20].  Figure 4 shows the pulsatile velocity profile which is dimensionless by the period of pulsatile cycle, *t*_*p*_, which is 0.8 s.

The fluid-structure interaction (FSI) conditions were used in the common boundary of fluid and solid. The governing equations for the solid-fluid coupled problem are as follows [21]:

(5) dfds:  Displacement,n·σf=n·σs:  Traction,d˙f=d˙s:  No slip,

where *d*, *σ*, and *n* are displacement, stress tensor, and normal vectors. The governing equations of the solid domain are as follows [21]:

(6) $$\begin{array}{c} \rho_{s}{\ddot{d}}_{s}=\nabla_{\circ }\cdot \sigma_{s}+\rho_{s}f_{s}, \end{array}$$

where *ρ*_*s*_ is the wall density, *σ*_*s*_ is the Cauchy stress tensor, *f*_*s*_ is the body force vector, and *d*_*s*_ is the wall displacement vector.

**Figure 5:**
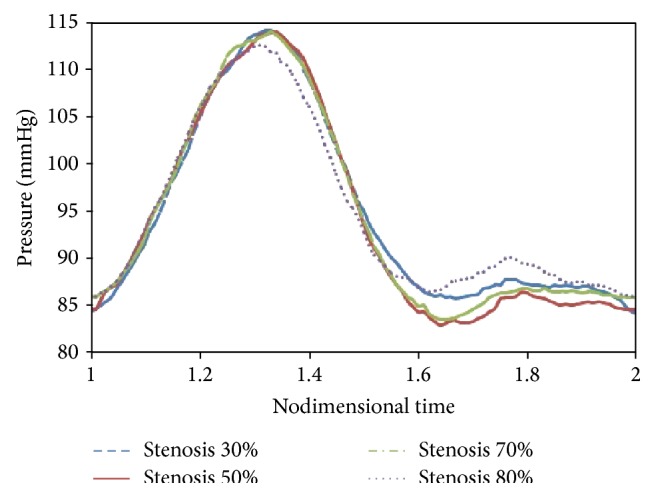
Outlet pressure pulse.

When studying the solid-fluid coupled problem, we should apply blood pressure pulse to the problem as the output condition. These pulses are obtained from experimental conditions and were shown in Figure 5 [21].

**Figure 6:**
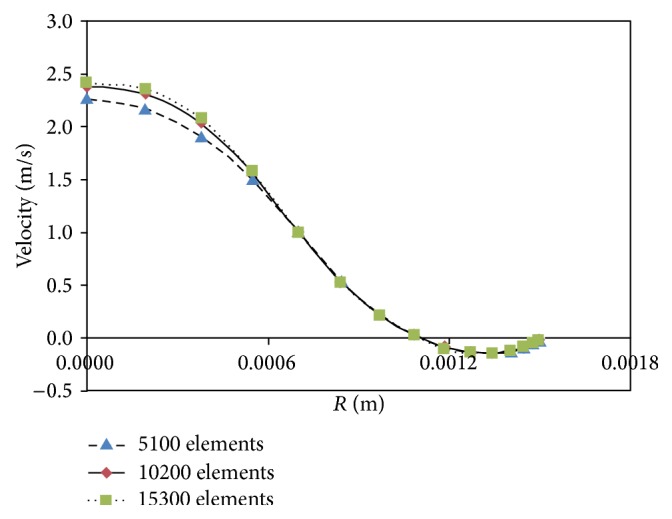
Results independency from grid of solution domain.

The axial velocity profiles at a distance of 1D from the stenosis throat in three types of meshing are shown in Figure 6. The results indicated that the results of 10200 computational cells and 15300 computational cells are consistent with each other. Thus, for reducing the computational time, 10200 computational cells will be used for calculations.

## 5. Results

**Figure 7:**
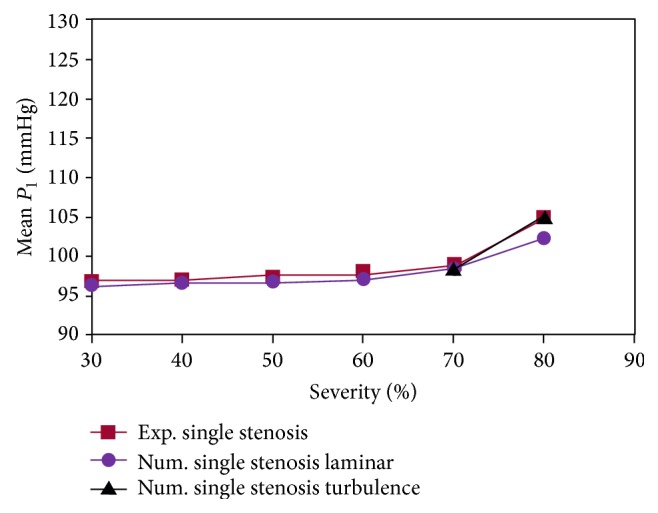
Comparisons between the inlet pressure.

Comparisons between the mean inlet pressure (*P*_1_) for stenosis percentages of 30%, 50%, 70%, and 80% are given in Figure 7. As can be seen, up to 70% stenosis, there is a very good consistency between experimental results [21] and the laminar flow assumption which suggests that, up to 70% stenosis, the flow is laminar. Shifting from 70% to 80%, the difference between experimental results and the laminar flow assumption increases, and there is a much higher consistency between experimental results and the turbulence flow assumption which suggests that, for 80% stenosis and higher, the flow is turbulent and the laminar flow assumption is not appropriate anymore. Mean inlet pressure for 80% stenosis in the case of laminar flow assumption is 102.4 mmHg, in the turbulent flow case is 105 mmHg, and in the experimental case is 104.8 mmHg. Given above, we select 80% stenosis and perform next calculations on it.

**Figure 8:**
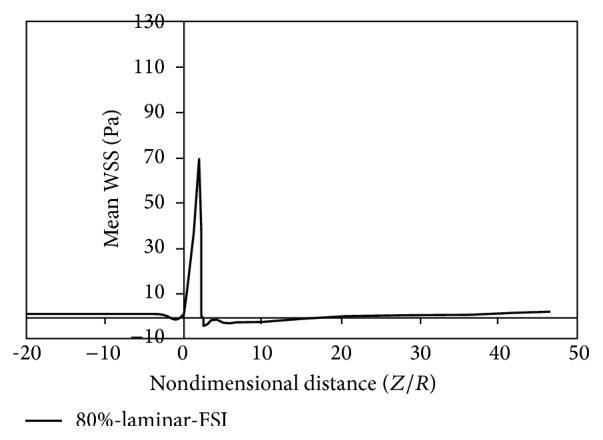
Mean WSS, laminar, flexible mode.

**Figure 9 fig9:**
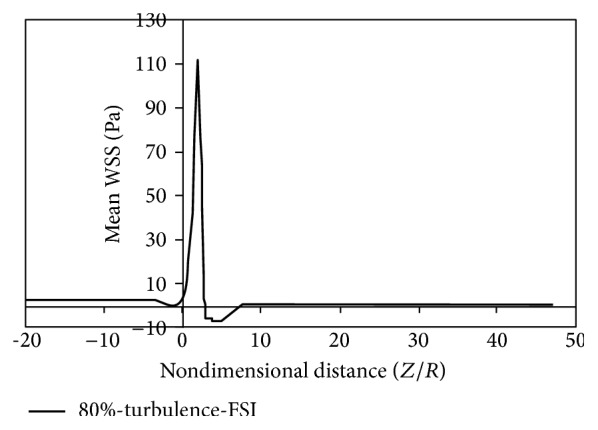
Mean WSS, turbulence, flexible mode.

**Figure 10 fig10:**
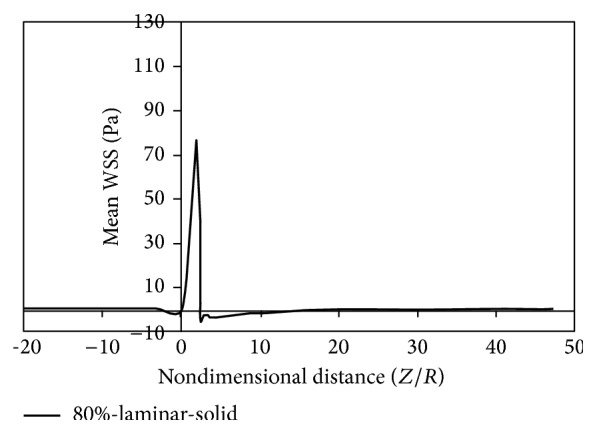
Mean WSS, laminar, and solid mode.

**Figure 11 fig11:**
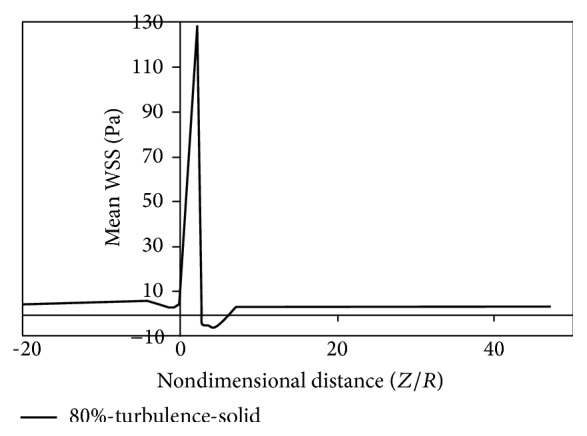
Mean WSS, turbulence, and solid mode.

**Figure 12 fig12:**
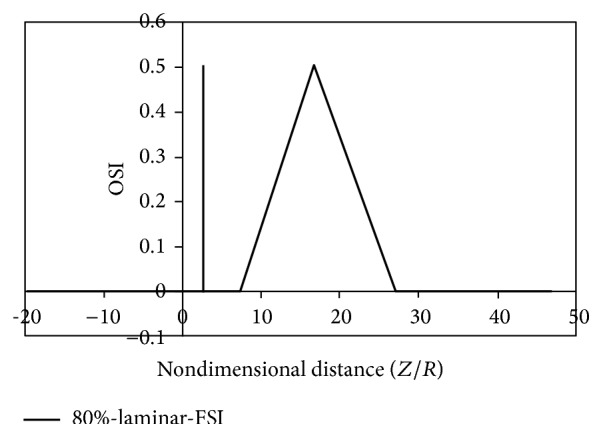
OSI, laminar, flexible mode.

**Figure 13 fig13:**
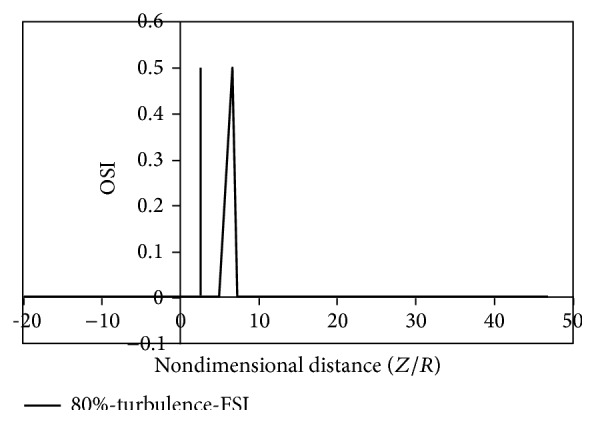
OSI, turbulence, flexible mode.

**Figure 14 fig14:**
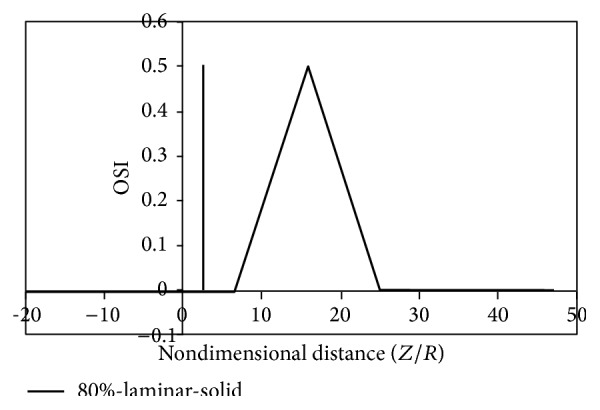
OSI, laminar, solid mode.

**Figure 15:**
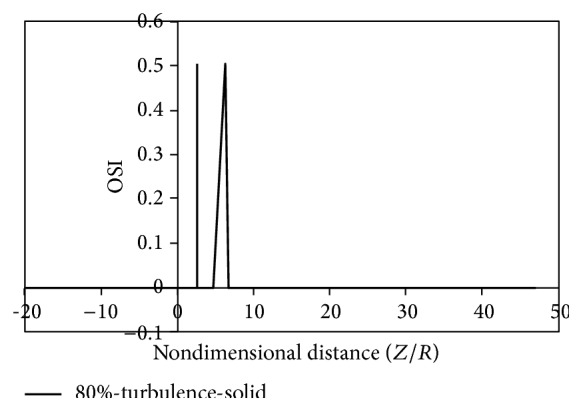
OSI, turbulence, solid mode.

Figures 8 [Fig fig9] [Fig fig10] [Fig fig11] [Fig fig12] [Fig fig13] [Fig fig14]to 15 show the timed-averaged changes of shear stress and the oscillatory shear index in the axial direction for 80% stenosis for the laminar or turbulent flow assumption and the rigid or flexible wall assumption. The time-averaged shear stress and the oscillatory shear index are among hemodynamic parameters used for identifying areas prone to arteriosclerosis.

The oscillatory shear index (OSI) is a mechanical parameter for flow oscillation showing the deviation of the wall shear stress from the dominant direction of blood flow during the cardiac cycle. The OSI value ranges from zero (for no change in the direction of wall shear stress) to 0.5 (for a 180-degree change in the direction of wall shear stress) [22]. To determine the OSI value, the following equation is used:

(7) $$\begin{array}{c} \text{OSI}=0.5\times \left(\;1-\frac{\left|\int_{0}^{T}\tau_{w}dt\right|}{\int_{0}^{T}\left|\tau_{w}\right|dt}\;\right). \end{array}$$

Time-averaged shear rate is defined as follows:

(8) $$\begin{array}{c} \text{Mean WSS}=\frac{1}{T}\overset{T}{\underset{0}{\int }}\tau_{w}dt. \end{array}$$

In the above equations, *T* is the periodicity of the cardiac cycle and *τ*_*w*_ is the shear stress vector.

As is clear from Figures 8 to 11, by changing from the flexible-wall mode to the rigid-wall mode as well as from the laminar flow assumption to the turbulent flow assumption, the time-averaged shear stress slightly increases in the prestenotic area. At the proximal shoulder region, the time-averaged shear stress significantly increases and, at the distal shoulder and poststenotic region, the time-averaged shear stress decreases further. This decrease in shear stress increases the production of reactive oxygen species and essentially increases the oxidation of LDLs in the intima. Oxidized LDLs stimulate endothelial cells to express leukocyte adhesion molecules such as vascular cell adhesion molecule-1 (VCAM-1) and intercellular adhesion molecule-1 (ICAM-1). Consequently, platelet adhesion to the endothelium and activation is possible, in an area where shear stress is low. Activated platelets release growth factors such as TGF-*β*. TGF-*β* significantly enhances proliferation of smooth muscle cells [23]. Studies have also shown that activated platelets release MMP-2, which mediates further platelet aggregation [24]. Thus poststenotic area not only is prone to develop plaque and new plaque formation, but also is more prone to the development of thrombosis. Angiographic studies have shown that plaque development occurs more in the poststenotic area [25] and the number of smooth muscle cells in the distal shoulder is far more than the proximal [26].

It can be seen from Figures 12 to 15 that there are two peaks for simple stenoses. In the simple stenosis, the first peak shows flow separation point and the second peak represents the reattachment point. Small values of time-averaged shear stress and high values of the oscillatory shear index both influence the cell secretion resulting in increased cell displacement and increased dissociation of intercellular junctions thereby increasing permeability of the LDL particles to the wall [27–29]. The experimental results of Deng et al. [30] also show high absorption of cholesterol in the flow reattachment point. Another thing that can be seen in Figures 12 to 15 is that for both the rigid artery wall mode and the flexible artery wall mode, by changing the laminar flow assumption to the turbulent flow assumption, the length of oscillatory zone highly decreases. This shows that, in high percentages of stenosis when using any of hemodynamic parameters, average shear stress, and oscillatory shear index for describing how the disease is developed, failure to consider the turbulent flow behavior can cause a large numerical error.

**Figure 16:**
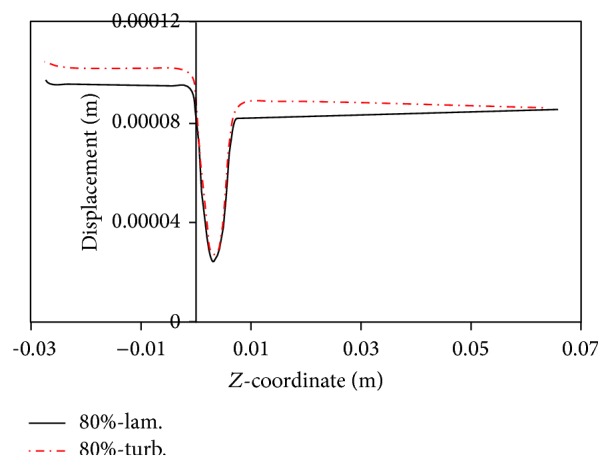
Radial displacement of wall at different mode.

Fry [31] stated that a shear stress over 40 Pa causes damage to endothelial cells. Ramstack et al. [32] stated that a shear stress greater than 100 Pa causes detachment of endothelial cells and clot formation. According to the contents of references [31, 32] and Figures 8–11 it can be seen that, in 80% stenosis with the laminar flow assumption, the endothelial cell operation is damaged. However, in 80% stenosis assuming turbulent flow, given that the maximum stress is greater than 100 Pa, the clot will form. Thus ignoring turbulence can make a different change in predicting damages. As can be seen in Figures 8 up to 15, the effect of turbulent flow on the maximum time-averaged shear stress of the wall on stenosis and the mean reverse flow area is more important than assuming flexible wall. The result that can be derived from Figure 16 is that the wall displacement with the turbulent flow assumption is more than the laminar flow assumption. Moreover, due to hypotension, the artery wall displacement in front of stenosis is more than the artery wall displacement at the back of stenoses.

**Figure 17:**
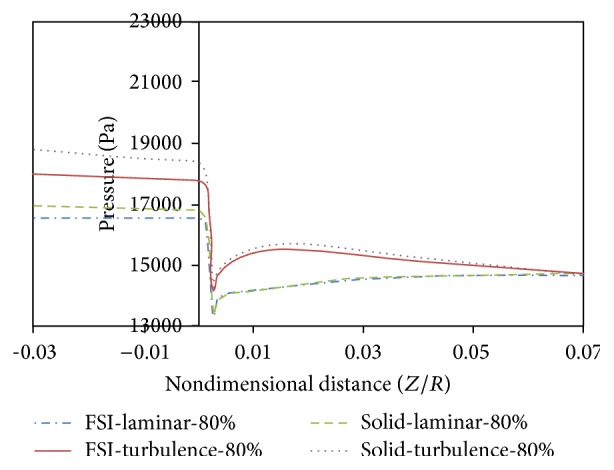
Changes in arterial pressure in the axial direction, *T*_2_.

Figure 17 shows changes in arterial pressure in the axial direction for simple stenosis with 80% stenosis at the maximum flow rate time. As can be seen from Figure 17, changing from flexible to rigid wall will increase the pressure in the proximal of stenosis. Moreover, changing from laminar to turbulent flow will increase the pressure in the proximal of stenosis. The hypotension in the turbulent mode is higher than the laminar mode and in the rigid wall artery mode higher than the flexible wall artery because of higher shear stress along the artery and consequently increased hypotension across the artery. Another result from Figure 17 is that, in front of the stenosis, the pressure difference at rigid and flexible modes is lower than at the back of stenosis. The reason is that, with decreasing pressure, the displacement of artery wall decreases and the artery wall becomes closer to the rigid mode.

**Figure 18:**
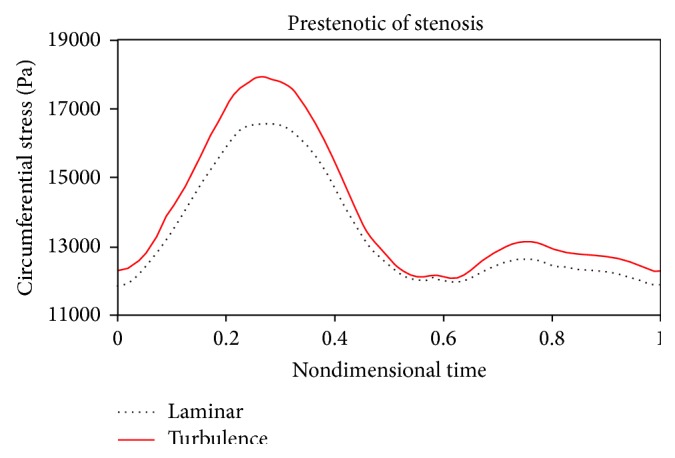
Circumferential stress, prestenotic.

**Figure 19 fig19:**
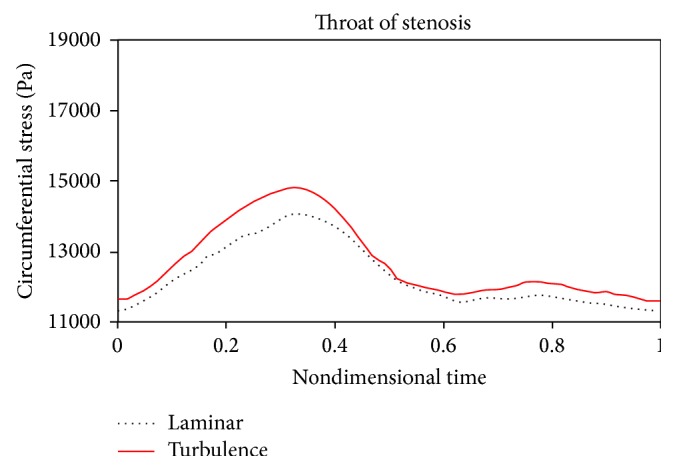
Circumferential stress, throat of stenosis.

**Figure 20:**
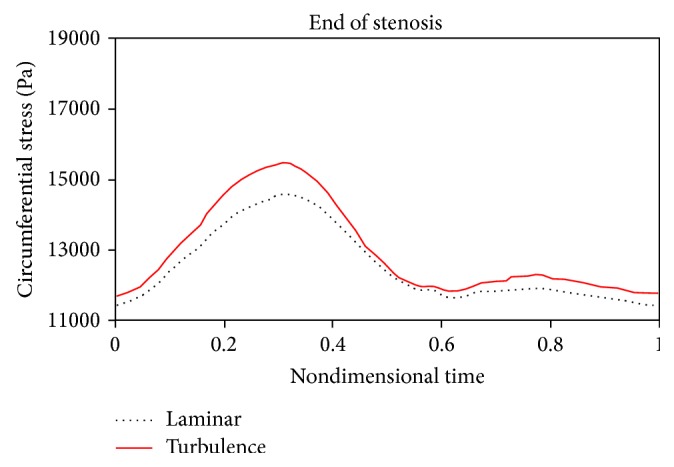
Circumferential stress, end of stenosis.

Figures 18 [Fig fig19]to 20 compare changes in circumferential stresses in time at different points for 80% stenosis. As can be seen, compared to the turbulent flow assumption, the laminar flow assumption shows lower circumferential stresses for the artery wall. The maximum circumferential stress is related to prestenotic zone because, according to Figure 17, the pressure exerted on the wall before the stenosis is higher. The minimum circumferential stresses are related to the stenosis peak because according to Figure 17, there is a sudden pressure drop due to serious tweaking. Another result from Figures 18 to 20 is that the laminar flow assumption at the stenosis peak shows circumferential stress lower than the turbulent flow assumption.

**Figure 21:**
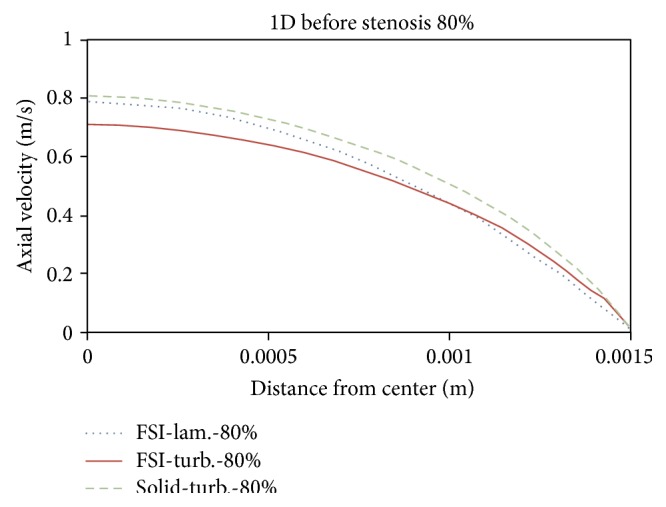
Axial velocity, 1D before stenosis.

**Figure 22 fig22:**
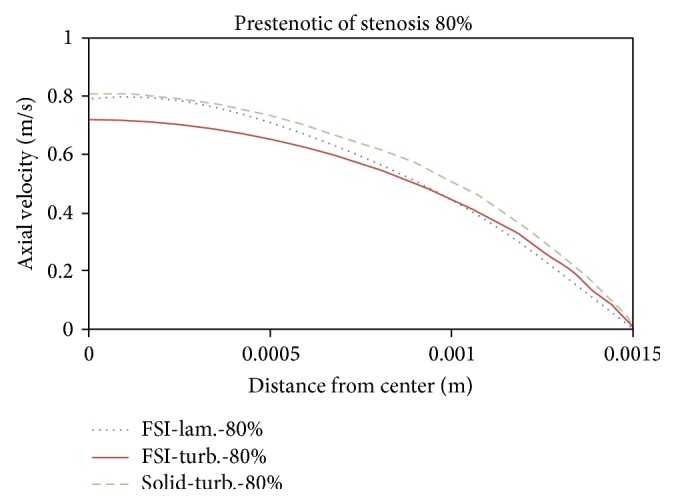
Axial velocity, prestenotic of stenosis.

**Figure 23 fig23:**
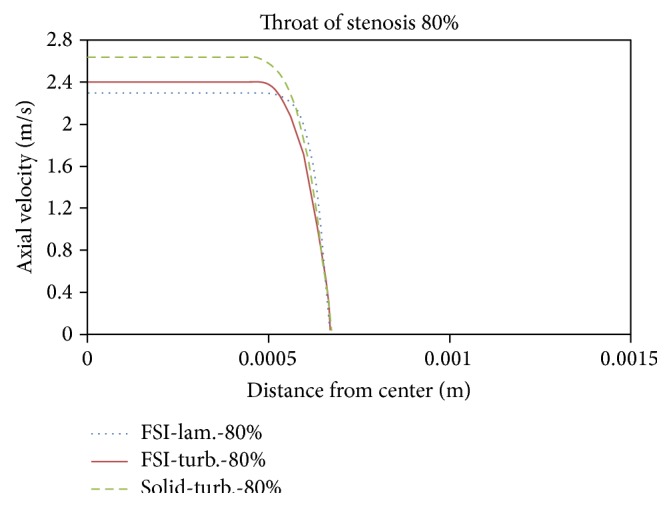
Axial velocity, throat of stenosis.

**Figure 24:**
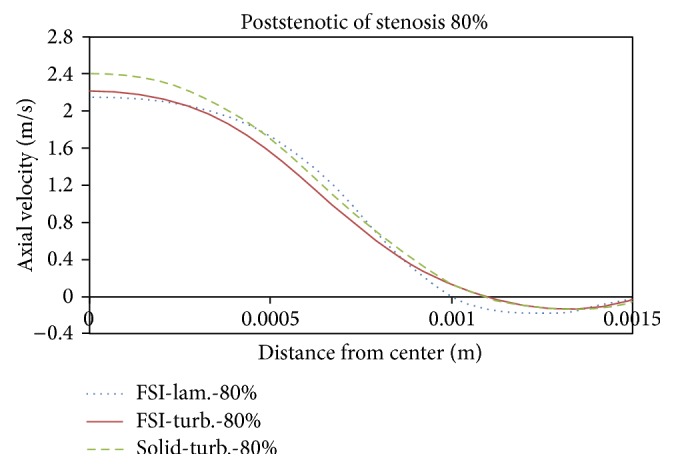
Axial velocity, poststenotic of stenosis.

Figures 21 [Fig fig22] [Fig fig23]to 24 show axial velocity profiles at the time of maximum flow rate (0.24 s), at a distance equal to the diameter before the stenosis, at the beginning of the stenosis, the throat, and the poststenotic of a simple 80% stenosis. Due to axial velocity profiles and also as expected the profile of turbulent flow assumption was obtained flatter than the laminar flow assumption, and the rigid wall mode further demonstrates maximum axial velocity.  Figure 23 shows velocity profiles at the throat of the simple stenosis. It shows that the maximum velocity reaches a value much higher than 1 m/s. This value is beyond a normal biological mode and may cause disturbances in the blood circulatory system. Also, as can be seen in Figure 24, at the end of stenosis compared with the throat of the stenoses, the maximum velocity is reduced and both the laminar flow assumption and the turbulent flow assumption at near of the wall predict the reverse flow. However, the laminar flow overestimates the reverse flow and shows the reverse flow zone larger which is obvious because the fluid has a lower energy and is soon detached from the surface. In other words, using the laminar flow assumption a wider zone at the back of stenosis is exposed to the disease and using the turbulent flow assumption the growth speed of plaques is higher.

**Figure 25:**
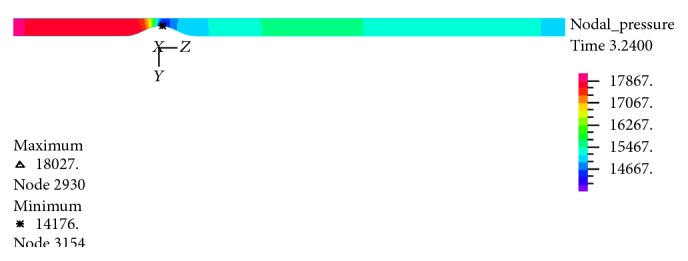
Pressure distribution at maximum flow rate *T*_2_, FSI-Turb.

**Figure 26:**
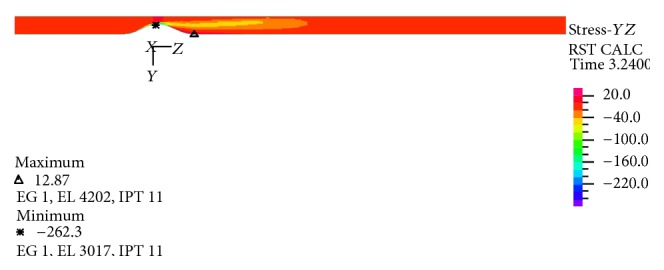
Shear stress distribution at maximum flow rate *T*_2_, FSI-Turb.

**Figure 27:**
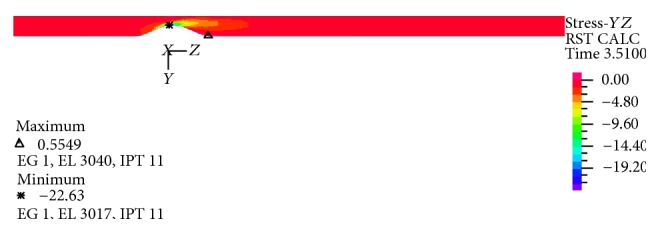
Shear stress distribution at minimum flow rate *T*_4_, FSI-Turb.

**Figure 28:**
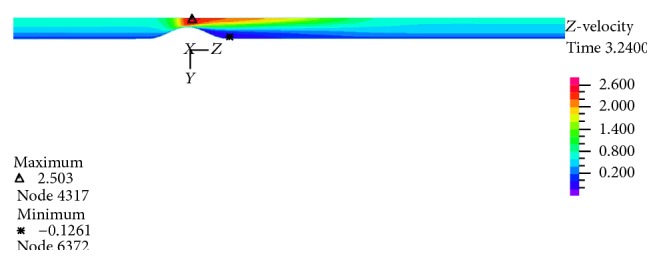
Velocity distribution at maximum flow rate *T*_2_, FSI-Turb.

In Figures 25–28 distribution of pressure, shear stress at *T*_2_ and *T*_4_ and axial velocity along the artery are shown, respectively. As seen, minimum pressure and maximum axial velocity occurred in the throat of stenosis. In distal of stenosis by reducing the velocity, the pressure is increased and Figure 17 also showed this. By away from stenosis region and reducing the effects of narrowing and opening of the flow cross section, pressure is reduced linearly. Blood shear stresses that exerted from the artery wall are shown in Figures 26 and 27. Because of high similarity between turbulent and FSI case study contour by other case studies, contours of other cases have been ignored. At the narrowest of cross section it can be seen from Figure 26 that shear stress increased suddenly and immediately after stenosis throat reduced severely and even negative values are seen. Then shear stress in the reverse flow region increased by mild slope and again gives positive value and remains constant until the end of the artery. As can be seen from Figures 26 and 27 the difference between the shear stress on the artery wall at maximum and minimum flow rate is very high and almost 11.6 times and, at other times of the cardiac cycle, shear stress on the artery wall has continuous changes. These frequent changes on the inner surface of the artery can lead to plaque rupture that can cause severe cramping and formation and the development of blood clot in the arteries. According to Figure 28, minimum axial velocity is −0.1261 m/s and occurred at the end of stenosis and maximum axial velocity is 2.503 m/s and occurred at the throat of stenosis.

## 6. Conclusion

Using ADINA software and taking into account the elastic wall and physiological pulses as a boundary condition, this paper assesses blood flow passing through the right coronary artery with a local stenosis. Compared with other numerical works, the *K*-*ε* standard model had a better consistency with the experimental work and was better able to show the reverse flow region. We therefore used the *K*-*ε* standard turbulence model to resolve the turbulent flow in the present numerical work. In the present work, the average inlet pressure for 80% stenosis in the laminar flow assumption was obtained 102.4 and at the turbulent flow assumption was obtained 105 compared with the other experimental works which was 104.8. This indicates that, for 80% stenosis, the flow is turbulent. As a result, 80% stenosis was selected as the sample stenosis. The effects of turbulent blood flow were examined on pressure drop and velocity profiles. The obtained results were compared with those of the laminar flow assumption and the rigid coronary artery wall. For both the rigid artery wall and the flexible artery wall, by changing from the laminar flow assumption to the turbulent flow assumption, the length of oscillatory region becomes much lower. This shows that in high percentages of stenosis when using any of the hemodynamic parameters of the average shear stress and the oscillatory shear index for describing how the disease is spread, failure to consider the turbulent flow behavior can cause a large numerical error. Another result of the present work is that, in 80% stenosis, the rigid artery wall assumption makes a smaller error compared to the laminar blood flow assumption.

## Figures and Tables

**Table 1:** Properties of fluid and artery wall.

Thickness of artery wall (m)	0.0005
Elasticity modulus of the artery wall (kPa)	910
Density of artery wall (Kg/m^3^)	1300
Poisson ratio of artery wall	0.49
Blood density (Kg/m^3^)	1050
Blood viscosity (Pa·s)	0.0033
